# Optimization of Internet of Things Remote Desktop Protocol for Low-Bandwidth Environments Using Convolutional Neural Networks

**DOI:** 10.3390/s24041208

**Published:** 2024-02-14

**Authors:** Hejun Wang, Kai Deng, Guoxin Zhong, Yubing Duan, Mingyong Yin, Fanzhi Meng, Yulong Wang

**Affiliations:** Institute of Computer Application, China Academy of Engineering Physics, Mianyang 621900, China; wanghejun1995@gmail.com (H.W.); dengkai@caep.cn (K.D.); zhoguoxin@126.com (G.Z.); duanyb@caep.cn (Y.D.); yinmy@caep.cn (M.Y.); mengfz@caep.cn (F.M.)

**Keywords:** Remote Frame Buffer protocol, IoT remote desktop, virtual network console, image compression

## Abstract

This paper discusses optimizing desktop image quality and bandwidth consumption in remote IoT GUI desktop scenarios. Remote desktop tools, which are crucial for work efficiency, typically employ image compression techniques to manage bandwidth. Although JPEG is widely used for its efficiency in eliminating redundancy, it can introduce quality loss with increased compression. Recently, deep learning-based compression techniques have emerged, challenging traditional methods like JPEG. This study introduces an optimized RFB (Remote Frame Buffer) protocol based on a convolutional neural network (CNN) image compression algorithm, focusing on human visual perception in desktop image processing. The improved RFB protocol proposed in this paper, compared to the unoptimized RFB protocol, can save 30–80% of bandwidth consumption and enhances remote desktop image quality, as evidenced by improved PSNR and MS-SSIM values between the remote desktop image and the original image, thus providing superior desktop image transmission quality.

## 1. Introduction

Remote desktops, which are essential in various sectors such as cloud computing and wireless sensor networks [1], face challenges with the growth of display resolutions. For instance, a 4K resolution desktop image, uncompressed, demands about 23.73 MB per frame. At 60 Hz, this equates to a bandwidth of roughly 1.39 GB/s. Such high bandwidth consumption can degrade user experience. VNC (Virtual Network Console), a popular remote desktop tool, uses the RFB (Remote Frame Buffer) protocol with its tight encoding mode employing JPEG for image compression. This approach conserves bandwidth. However, JPEG’s fixed parameters in its discrete cosine transform may not always suit the image’s features. Moreover, its block processing can introduce block effects and artifacts.

Some researchers have proposed different solutions for desktop image processing methods in remote desktop application scenarios. Lin et al. proposed a composite image compression algorithm specifically for remote desktop image transmission [2]. This algorithm uses a method similar to the block partitioning of desktop images in the JPEG compression algorithm. On this basis, the algorithm divides the text and graphics in the blocks and uses different compression methods for them than for images. For text and graphics, the color values can be indexed and compressed based on the index for text and graphics areas, while for areas with complex color information such as images, the JPEG compression algorithm is used for compression. However, this method still does not solve the shadow and blur problems caused by the JPEG compression algorithm for desktop images under low-quality parameters. Wang et al. proposed a composite image compression method and joint coding (UC) based on several effective text/graphics and natural image compression algorithms [3,4]. This method mainly uses lossy intra-frame hybrid coding tools or their variants to compress natural images, while for text/graphics, it mainly uses dictionary entropy coding, run-length coding (RLE) in the RFB protocol, Hextile in the RFB protocol, and PNG and other encoding tools as candidates for text/graphics compression. By appropriately combining these lossless tools with intra-frame hybrid coding, high compression performance is achieved for the text/graphics part of the composite image. This method uses an optimizer based on rate–distortion cost to separate natural images and text/graphics, and finally selects lossy and lossless coding tools for encoding, respectively. However, this desktop image coding scheme still has some drawbacks. Firstly, the image to be encoded needs to be divided into small pixel blocks. Secondly, iterative selection is required for different block coding, which may affect the real-time performance of image coding. Lastly, the support for high-resolution images is not good enough, which cannot adapt well to the current era of high-resolution desktop images.

Several researchers have optimized desktop image coding methods for specific remote desktop usage scenarios. Sazawa et al. proposed a desktop image coding scheme for engineering applications such as 2D-CAD and CAE: Remote Virtual Environment Computing (RVEC) [5]. RVEC combines movie compression and compression algorithms with small static image loss, which greatly reduces the bandwidth of image transmission without compromising the acceptable engineering standards. In addition, RVEC uses a new lossless compression algorithm, the graphic compression algorithm, to compress static images applied to graphics. The graphic compression algorithm uses vector features in the displayed image to obtain better compression ratios without affecting the compression speed. Shimada et al. later proposed a high-compression method for graphic images in 3D-CAD, CAE software, and other engineering applications based on Sazawa’s research [6]. This method uses the characteristic that the pixel values in artificial images do not change locally and extracts constant gradients through frequency transformation, fully utilizing the characteristics of graphic images. It provides reasonable actual compression time, compression size, and image quality for engineering applications in cloud environments. However, although the image coding scheme proposed by Sazawa, Shimada, and others performs well in the field of engineering applications, it is not universal and therefore difficult to apply to general remote desktop usage scenarios.

Deep learning’s advancements [7] offer promising applications in image processing. Its ability to discern image features can reduce data redundancy, optimizing bandwidth for remote desktop transmission. Given that users focus on specific desktop areas, deep learning can enhance the reconstruction of text and graphics, improving visual experience.

Deep learning-based image compression methods have been proposed, showing performance approaching or surpassing traditional methods [8]. This potential paves the way for its application in remote desktop image transmission. This paper focuses on constructing an algorithm that is more suitable for remote desktops, replicating the method proposed by Ballé et al. [9], with Mean Squared Error (MSE) as the optimization target. Despite optimizing Peak Signal-to-Noise Ratio (PSNR) and Structural Similarity (SSIM) of images [10], this method presents some limitations [11]. Considering the importance of users’ subjective perception of desktop image quality in performance evaluation, this paper introduces Multi-Scale Structural Similarity (MS-SSIM) as an additional optimization target. Moreover, an adaptive attention mechanism module is introduced, combining spatial and channel attention, which serves as an image encoder enhancement module, applying attention weights to the latent representations of images [12,13,14,15]. Subsequently, this paper improves the existing RFB protocol based on this image compression algorithm, introducing a novel encoding method. This approach employs a convolutional neural network-based image compression technique as its core method for encoding images. Experimental results indicate that the enhanced RFB protocol proposed in this paper can reduce the bandwidth consumption for remote desktop usage by 30–80% without compromising the adequacy of remote desktop image transmission.

Compared to the preliminary conference version of the paper [16], this work has expanded on it by designing protocols based on the input–output characteristics of the CNN model to enable its application in remote desktops. In this work, several different processing approaches are introduced to maximize the improvement in image transmission efficiency of the optimized RFB protocol, while ensuring a balance between bandwidth consumption and hardware performance. Ultimately, this paper demonstrates the effectiveness of our proposed optimized RFB protocol—significantly reducing bandwidth consumption in remote desktops while enhancing the quality of transmitted desktop images.

The remainder of this paper is organized as follows: Section 2 reviews related work, while Section 3 analyzes the differences between various image quality assessment methods. Section 4 introduces the image compression techniques employed in the improved RFB protocol proposed in this paper. Section 5 details the proposed improved RFB protocol. Section 6 presents the experimental results and analysis, and Section 7 offers conclusions.

## 2. Related Works

In the domain of remote desktop technologies, significant progress has been made in optimizing the performance and efficiency of transmission protocols, particularly focusing on encoding strategies and virtualization enhancements. A notable contribution in this field is the work by Halim [17], which presents a detailed framework for evaluating the encoding performance in remote desktop systems, with a specific focus on the TigerVNC protocol. This analysis is crucial as it provides insights into the optimization of image transmission, which is a key factor in remote desktop interactions. Complementing this, the study by Li et al. [18] investigates the enhancement of service transportation in cloud-based virtual desktop infrastructures. Their research sheds light on the integral role of video encoding and compression algorithms in improving the overall efficiency and performance of virtual desktop systems. Both studies collectively offer a comprehensive understanding of the current advancements and challenges in remote desktop protocol technologies, underlining the importance of continuous innovation in this rapidly evolving field.

Most image compression methods based on deep learning are lossy image compression methods [19]. Due to the robust modeling capabilities of deep learning, these algorithms have gradually approached or even surpassed the performance of traditional image compression methods. Currently, the primary deep learning-based image compression techniques involve the integration of convolutional neural networks (CNNs), recurrent neural networks (RNNs), and generative adversarial networks (GANs). CNNs, in particular, have seen rapid development in image processing, excelling in tasks such as object detection, image classification, and semantic segmentation. The sparse connectivity and parameter sharing properties of CNN convolution operations have demonstrated advantages in image compression.

In 2016, Ballé et al. [20] introduced a parametric nonlinear model for Gaussianizing data derived from natural images. Their primary contribution was the development of a normalization layer—the Generalized Divisive Normalization (GDN) layer—optimized for image compression and reconstruction tasks. This layer effectively reduces random noise introduced by traditional Batch Normalization (BN) layers. Later, Ballé et al. [21] proposed an image compression technique based on nonlinear transformation and uniform quantization. This approach leverages a CNN to extract latent feature representations from images and implements local gain via the previously proposed GDN layer to reduce additive noise. This marked the first integration of CNNs with image compression techniques, establishing a foundation for the development of end-to-end CNN-based image compression methods. Subsequently, Ballé et al. [9] introduced an end-to-end image compression model that integrates hyperprior encoders with decoders. This model effectively captures spatial dependencies within latent feature representations and eliminates redundancy via Gaussian distribution modeling, resulting in lower bit-rate image compression. Jiang et al. [22] introduced an end-to-end CNN-based image compression encoder–decoder. This approach utilizes a neural network in conjunction with traditional image processing techniques to process images. Initially, a CNN-based image encoder is employed to extract a compact representation of the image, effectively shrinking the original image along its height and width dimensions. Subsequently, traditional image compression methods (e.g., JPEG or BPG) are utilized to encode this compact representation of the image. At the decoding stage, traditional image compression encoding is first applied to restore the compact representation of the image. Then, a CNN-based image decoder is used to scale up this compact representation and restore it back into its original form as an image. Zhao et al. [23] argued that integrating traditional forms of image compression may not be optimal for deep learning-based approaches to this task. As such, they proposed a method that employs a virtual encoder to connect the encoding and decoding stages during training. This virtual encoder—also based on CNNs—can directly map compact representations onto image code streams transmitted to the decoding stage via nonlinear mapping. This approach effectively links neural network-based encoding and decoding stages and can be integrated with traditional encoders to produce high-quality reconstructed images. It can also be extended to other end-to-end CNN-based image compression architectures. To enhance the quality of reconstructed images, Liu et al. [22] introduced a decoding stage enhancement module capable of learning residual information between reconstructed and original images via neural networks. By leveraging this residual information to improve decoding performance, the module further enhances the quality of images reconstructed by decoders.

## 3. Algorithm for Remote Desktop Image and Compression Quality Evaluation

Image compression algorithms are typically evaluated by applying image quality standards to measure the extent of quality degradation after compression. These evaluation techniques can be categorized into subjective and objective assessments. Subjective assessment involves viewer-based scoring, wherein several evaluators grade the reconstructed image against the original one, with the mean score serving as the final evaluation. In contrast, objective assessment calculates the differences between images using mathematical models. Although subjective assessments are time-consuming and affected by numerous factors, objective assessments offer automated scoring that is not influenced by viewer bias.

Common objective image quality metrics include Peak Signal-to-Noise Ratio (PSNR), which is widely used in the field of image and video processing. PSNR, calculated conveniently through Mean Squared Error (MSE), quantifies image distortion. The computation equation for MSE is presented in Equation (1), and substituting it into Equation (2) gives the PSNR value for an image, where n represents the number of pixels in the image.

(1) $$\mathrm{MSE}=\frac{1}{n}\sum_{i=1}^{n}{\left(x_{i}-{\hat{x}}_{i}\right)}^{2}$$

(2) $$\mathrm{PSNR}=10\times \log_{10}\left(\frac{{\left(2^{n}-1\right)}^{2}}{MSE}\right)$$

In optimizing the quality of reconstructed desktop images, the Mean Squared Error (MSE) method presents certain limitations. MSE, offering equal weight to all pixels, may not aptly assess reconstructed desktop images. Even with identical MSEs, perceptual differences between images can be significant, indicating that the Peak Signal-to-Noise Ratio (PSNR) derived from MSE may not accurately represent human perception. When viewing desktop images, the human eye often concentrates on specific work areas and is more sensitive to noise in these sections than in other areas. Furthermore, the human eye has greater sensitivity to luminance than color, a facet overlooked when merely calculating MSE between the reconstructed image and the original. In contrast, the Structural Similarity Index (SSIM) estimates luminance similarity, contrast, and structure similarity between two images by calculating their means, variance, and covariance. Equations (3), (4) and (5), respectively, represent the calculations for luminance similarity, contrast, and structure similarity.

(3) $$l\left(x,y\right)=\frac{2\mu_{x}\mu_{y}+c_{1}}{\mu_{x}^{2}+\mu_{y}^{2}+c_{1}}$$

(4) $$c\left(x,y\right)=\frac{2\sigma_{x}\sigma_{y}+c_{2}}{\sigma_{x}^{2}+\sigma_{y}^{2}+c_{2}}$$

(5) $$s\left(x,y\right)=\frac{\sigma_{xy}+c_{3}}{\sigma_{x}\sigma_{y}+c_{3}}$$

Here, $C_{1}={\left(K_{1}L\right)}^{2}$, $C_{2}={\left(K_{2}L\right)}^{2}$, $C_{3}=\frac{C_{2}}{2}$ with $K_{1}=0.01$, $K_{2}=0.03$, and $L=2^{B}-1$, where *B* denotes the pixel depth of the image. The structural similarity between the distorted and original images can be calculated by substituting Equations (3)–(5) into Equation (6), with α=β=γ=1. Optimal performance of structural similarity requires specific configuration. In contrast, Multi-Scale Structural Similarity (MS-SSIM) calculates the degree of structural similarity at different scales between the distorted and original images through multiple low-pass filtering and downsampling processes, maintaining good performance across different image resolutions. Equation (7) describes the calculation of structural similarity at multiple scales, where $\alpha_{j}=\beta_{j}=\gamma_{j}$ and $\sum_{j=1}^{M}\gamma_{j}=1$.

(6) $$\mathrm{SSIM}\left(x,y\right)={\left[l\left(x,y\right)\right]}^{\alpha }{\left[c\left(x,y\right)\right]}^{\beta }{\left[s\left(x,y\right)\right]}^{\gamma }$$

This study aims to optimize the performance of end-to-end image compression models using Mean Squared Error (MSE) combined with Multi-Scale Structural Similarity (MS-SSIM) as the loss function. By disregarding the quality of monotonic or non-working areas that are less relevant to human perception, we ensure a more complete preservation of image information in the working area of the reconstructed desktop image, thereby enhancing the comprehensive performance of the proposed method in the field of desktop image compression.

(7) $$\mathrm{SSIM}\left(x,y\right)={\left[l_{M}\left(x,y\right)\right]}^{\alpha }M\prod_{j=1}^{M}{\left[c_{j}\left(x,y\right)\right]}^{\beta_{j}}{\left[s_{j}\left(x,y\right)\right]}^{\gamma_{j}}$$

## 4. Convolutional Neural Network-Based Image Compression Codec

### 4.1. Overview

**Figure 1:**
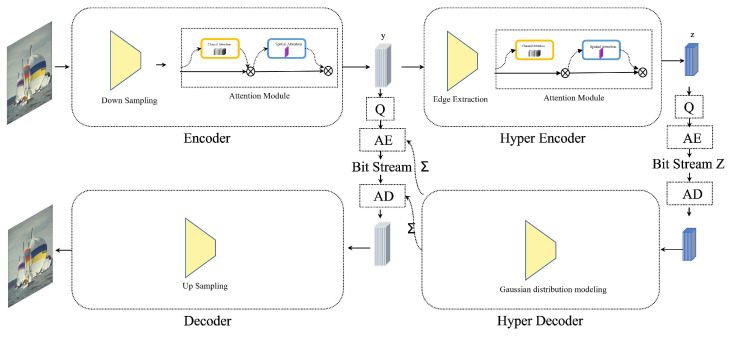
The architecture of proposed end-to-end image compression model.

As depicted in Figure 1, the proposed model includes an image encoder, image decoder, and a hyperprior encoder–decoder. The input digital image *x* is transformed into latent representation *y* via the encoding network and then quantized to $\hat{y}$ by the quantizer *Q*. In the decoding stage, $\hat{y}$ is used to reconstruct image $\hat{x}$. The hyperprior encoder–decoder extracts edge information from the latent representation, reducing redundancy and facilitating the entropy coding process, thereby achieving a shorter coding length and higher compression ratio.

The encoder, primarily comprising a downsampling module and a spatial channel attention module, extracts latent feature representation. Under the attention module, the final latent feature representation *y* is obtained with applied channel and spatial attention.

The downsampling module consists of four convolutional layers and a Generalized Divisive Normalization (GDN) layer between each pair. It extracts the latent feature representation by downsampling the input image *x*. If a higher compression rate is required at some quality loss, 128 convolution kernels per convolutional layer are sufficient. For higher-quality reconstructed images, 192 convolution kernels per layer are needed.

Between each pair of convolutional layers, a normalization layer, the GDN, is applied. While Batch Normalization (BN) is typically used in deep learning-based image processing tasks, in this study, the advantages of BN become disadvantages. Hence, BN is unsuitable for this application. Conversely, the GDN layer, which is more appropriate for image reconstruction, eliminates the additive noise brought by BN. During training, the GDN layer normalizes the feature map through unsupervised learning, performing Gaussianization on the symbolic data. The GDN layer is expressed as in Equations (2)–(8).

(8) $$y_{i}=\frac{x_{i}}{{\left(\beta_{i}+\sum_{i}\gamma_{i}\times x_{i}^{2}\right)}^{\frac{1}{2}}}$$

In the quantization stage, this paper employs Equations (9) and (10) to quantize the latent representation *y* into $\hat{y}$. This approach adds uniform additive noise to the latent feature representation instead of directly truncating the fractional part, ensuring quantization of the feature representation while preserving gradients.

(9) $$\hat{y}=y+\Delta y$$

(10) Δy∼μ(−0.5,0.5)

The decoder utilizes quantized latent features as input, reconstructing the image through a number of transposed convolutional layers mirroring the encoder. Most layers match the encoder’s convolutional kernel size, quantity, and stride: 128 5 × 5 kernels with a stride of 2 under low-quality encoding, and 192 5 × 5 kernels with the same stride under high-quality conditions. An exception is the final layer, utilizing three 5 × 5 kernels to reconstruct the image’s three color channels. Between every two transposed convolutional layers in the decoder, an “Inversed Generalized Divisive Normalization” (IGDN) layer is inserted to reverse the GDN layer’s output, assisting image reconstruction. The calculation process for the IGDN layer aligns with Equation (11).

(11) $$y_{i}=x_{i}\times {\left(\beta_{i}+\sum_{i}\gamma_{i}\times x_{i}^{2}\right)}^{\frac{1}{2}}$$

The hyperprior encoder $h_{a}$ extracts edge information from latent features *y*, yielding edge representation *z*. This is quantized to $\hat{z}$, aiding encoding of *y*. The hyperprior decoder $h_{s}$ uses $\hat{z}$ to estimate the standard deviation distribution $\hat{\sigma }$ of $\hat{y}$ and encode it. $\hat{z}$, which is also needed at the decoding stage for $\hat{\sigma }$ estimation and entropy decoding, must be compressed and transmitted from the encoder to the decoder. Edge information is extracted from *y* via a convolutional layer and a nonlinear activation function in $h_{a}$, while $h_{s}$ uses a transposed convolution and a nonlinear activation function to estimate $\hat{\sigma }$. The standard deviation $\hat{\sigma }$ models $\hat{y}$ using a Gaussian distribution (Equation (12)). Substituting Equation (12) and $\hat{\sigma }$ into Equation (13) yields the probability distribution of any symbol in $\hat{y}$. During model training iterations, symbols with minimal impact on image quality approach zero, increasing their probability and shortening the encoding length during entropy coding, thus removing redundancies.

(12) $$N\left(x|\mu ,\sigma \right)=\frac{1}{\sqrt{2\pi }\sigma }\exp\left(-\frac{{\left(x-\mu \right)}^{2}}{2\sigma^{2}}\right)$$

(13) $$p_{\hat{y_{i}}}\left(\hat{y_{i}}|\hat{\sigma_{i}}\right)=\int_{\hat{y_{i}}-\frac{1}{2}}^{\hat{y_{i}}+\frac{1}{2}}N\left(\hat{y_{i}}|0,\hat{\sigma_{i}}\right)dy$$

### 4.2. Attention Mechanism

**Figure 2:**
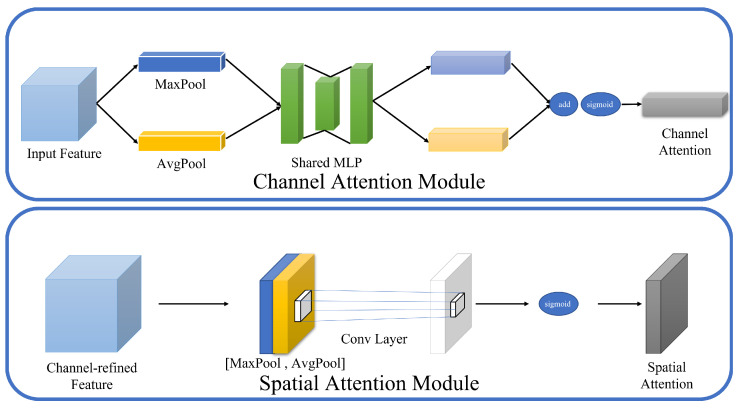
Attention mechanism.

This paper introduces the Convolutional Block Attention Module (CBAM) [24], which is a mechanism that combines spatial and channel attention to adaptively learn the importance of different channels and spatial positions within feature maps. By weighting feature maps in multiple dimensions, it achieves improved compression and reconstruction. Its detailed structure is presented in Figure 2.

For channel attention, CBAM initially extracts weights from different channels within feature maps through max pooling and average pooling. Subsequently, it applies two fully connected layers to perform nonlinear transformations on these pooled results. To obtain the final channel weights, it first sums the output from the fully connected layers and then applies another nonlinear transformation. The input to this operation is $F_{c_{in}}\in R^{C\times H\times W}$, with the computation process as shown in Equations (14) and (15).

(14) $$M_{c}\left(F\right)=\sigma \left(\mathrm{FC}\left(\mathrm{AvgPool}\left(F\right)\right)+\mathrm{FC}\left(\mathrm{MaxPool}\left(F\right)\right)\right)$$

(15) $$\mathrm{FC}\left(F\right)=W_{1}\left(\sigma \left(W_{0}\left(F\right)\right)\right)$$

In terms of spatial attention, the input feature map undergoes max pooling and average pooling along the channel axis, thereby converting the input dimensions from $F_{s_{in}}\in R^{C\times H\times W}$ to 2×H×W. A convolution operation is then performed on this dimensionally transformed feature map to extract weights from different spaces, as shown in Equation (16). Finally, the effects of channel attention and spatial attention are collectively applied to the feature map, as described by Equation (17).

(16) $$M_{s}\left(F\right)=\sigma \left(\mathrm{Conv}\left(\theta ,\left[\mathrm{AvgPool}\left(F\right);\mathrm{MaxPool}\left(F\right)\right]\right)\right)$$

(17) $$F^{\prime }=M_{s}\left(M_{c}\left(F\right)⊗F\right)⊗\left(M_{c}\left(F\right)⊗F\right)$$

### 4.3. Loss Function

The end-to-end image compression model balances distortion and bitrate according to Equation (18), where λ—analogous to the quality parameter QP in JPEG—controls reconstructed image quality and storage space requirements. The distortion function, d(·), quantifies the distortion between the input and reconstructed image (Equation (19)), considering both MS-SSIM ($L_{SSIM}$, Equation (20)) and L2 error ($L_{MSE}$, Equation (21)) with parameter α balancing attention to these aspects. The rate estimation function, H(·), includes the bitrates required for encoding the feature representation $\hat{y}$ and the auxiliary information $\hat{z}$ (Equations (22) and (23)). The probability distribution function for $\hat{z}$ and its cumulative distribution function are given in Equations (24)–(28), with parameters *a*, *h*, and *b* following a normal distribution N(0,0.01), and *K* is set to 4 in this study.

(18) $$L=\lambda D+R=\lambda d\left(\hat{x},x\right)+H\left(\hat{y}\right)+H\left(\hat{z}\right)$$

(19) $$d\left(\hat{x},x\right)=\alpha \times L_{SSIM}\left(\hat{x},x\right)+\left(1-\alpha \right)\times L_{MSE}\left(\hat{x},x\right)$$

(20) $$L_{SSIM}=1-SSIM\left(\hat{x},x\right)$$

(21) $$L_{MSE}=\frac{1}{N}\sum_{i=1}^{N}\left|\right|{\hat{x}}_{i}-x_{i}{\left|\right|}^{2}$$

(22) $$H\left(\hat{y}\right)=-\sum_{i}\log_{2}P_{\hat{y}}\left(\hat{y}|\hat{z}\right)$$

(23) $$H\left(\hat{z}\right)=-\sum_{i}\log_{2}P_{\hat{z}}\left(\hat{z}\right)$$

(24) $$p_{{\hat{z}}_{i}}\left({\hat{z}}_{i}\right)=\mathrm{BitEstimator}\left({\hat{z}}_{i}+\frac{1}{2}\right)-\mathrm{BitEstimator}\left({\hat{z}}_{i}-\frac{1}{2}\right)$$

(25) $$\mathrm{BitEstimator}\left(x\right)=f_{K}\circ f_{K-1}\circ \ldots \circ f_{1}=f_{K}\left(f_{K-1}\left(\ldots f_{1}\left(x\right)\right)\right)$$

(26) $$f_{K}\left(x\right)=\sigma \left(x⊙\zeta \left(h\right)⊕b\right)$$

(27) $$f_{k}\left(x\right)=g_{k}\left(x⊙\zeta \left(h\right)⊕b\right)$$

(28) $$g_{k}\left(x\right)=x⊕\tanh\left(x\right)⊙\tanh\left(a\right)$$

## 5. Optimized RFB Protocol

### 5.1. The Expansion of the RFB Protocol

The open-source version of the RFB protocol currently supports nine different image encoding and transmission methods, as detailed in Table 1. Raw encoding transmits the original pixel data of the requested update area directly, scanning from left to right and top to bottom. Building on Raw, CopyRect sends the positional data of areas on the desktop that have changed in location but not content. CoRRE, a variant of RRE, inherits the characteristics of RRE and is capable of encoding and transmitting foreground and background colors separately, making it particularly effective for screens with monochromatic colors. ZRLE, zlib, and Tight, on the other hand, employ various compression algorithms to compress data for transmission or compress the original desktop images before transmission. We have expanded the RFB protocol based on its open-source version 3.8, registering a new method of desktop image transmission encoding. This encoding, assigned the number 24, is referred to in this paper as DeepTight encoding.

### 5.2. Research on Encoding Schemes

The RFB protocol updates the screen by only refreshing the parts that have changed, dividing these altered sections into rectangles of varying sizes for encoded transmission. The encoding scheme for the pixels within these sub-rectangles varies depending on the encoding method and pixel arrangement. This paper reviews the implementation of existing encoding schemes in the RFB protocol and designs the DeepTight encoding, which is tailored to the characteristics of image compression and decompression codecs based on deep learning.

#### 5.2.1. Partitioning of Update Regions

To balance the data volume of single encoded images with the number of encoding iterations for the rectangles pending update, this study imposes restrictions on the size of each encoding area. The specific rules are as follows: the height or width of a single area to be encoded must not exceed 480 pixels, and the total number of pixels in a single rectangular area to be encoded should not exceed 320 pixels by 320 pixels. The algorithm for dividing sub-rectangles is as follows (Algorithm 1):
**Algorithm 1** Update Region Dividing**Require:** The left-top pixel location (left,top) and the right-bottom pixel location (right,bottom) of an update region;**Ensure:** A queue of sub-rectangles;  1:subRectangles←queue()  2:w←right−left  3:h←bottom−top  4:**if** w≤480 and h≤480 and w×h≤320×320 **then**  5:    subRectangles.push(region)  6:    **return** subRectangles  7:**else**  8:    $x_{num}\leftarrow \mathrm{round}\left(\left(w+320-1\right)/320\right)$  9:    $y_{num}\leftarrow \mathrm{round}\left(\left(h+320-1\right)/320\right)$10:   $x_{step}\leftarrow \mathrm{round}\left(w/x_{num}\right)$11:   $y_{step}\leftarrow \mathrm{round}\left(h/y_{num}\right)$12:   **for** j=0 to $y_{num}$ **do**13:      $top_{subrectangle}\leftarrow top+j\times y_{step}$14:      $bottom_{subrectangle}\leftarrow \left(j=y_{num}-1\right)?bottom:\left(top_{subrectangle}+y_{step}\right)$15:      **for** i=0 to $x_{num}$ **do**16:         $left_{subrectangle}\leftarrow left+i\times x_{step}$17:         $right_{subrectangle}\leftarrow \left(i=x_{num}-1\right)?right:\left(left_{subrectangle}+x_{step}\right)$18:         subRectangles.push(subrectangle)19:      **end for**20:   **end for**21:   **return** subRectangles22:**end if**

#### 5.2.2. Identification of Monochromatic Update Regions and Handling of Minor Update Areas

**Figure 3:**
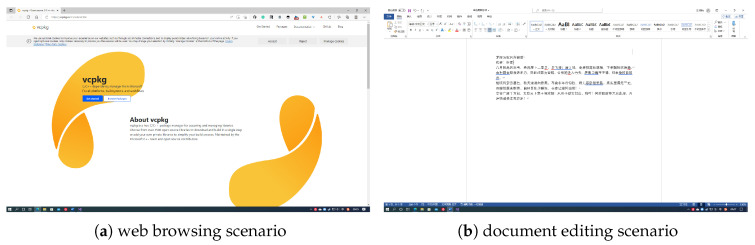
Different usage scenarios of remote desktops.

Figure 3 illustrates two real-world scenarios of user interface usage: web browsing and document editing. As shown in the figure, apart from the areas displaying web pages and document content, there is a significant amount of blank space, which constitutes monochromatic regions. In most current remote desktop usage scenarios, these monochromatic areas occupy a substantial proportion.

The redefined areas awaiting updates often contain several monochromatic sub-rectangles. Correctly identifying and processing these monochromatic sub-rectangles, particularly the efficient handling of such areas, can enable the client to bypass the decoding step and directly fill these regions. This approach significantly reduces data transmission for remote desktops and accelerates the encoding and decoding speed of desktop images between the server and the client. The algorithm for identifying monochromatic sub-rectangles in this study is as follows (Algorithm 2):
**Algorithm 2** Solid Rectangle Recognizing**Require:** The pixels of a rectangle with its left-top pixel location (left,top) and its right-bottom pixel location (right,bottom);**Ensure:** If a rectangle is a solid rectangle;  1:pix←rectangle[left,top]  2:**for** j←top to bottom **do**  3:   **for** i←left to right **do**  4:      **if** pix≠rectangle[i,j] **then**  5:         **return** False  6:      **end if**  7:   **end if**  8:**end for**  9:**return** True

In addition, this study also addresses the potential presence of tiny, non-monochromatic areas awaiting update. The DeepTight encoding considers any update area with a total pixel count less than 100 as a minor update area. If this area is not monochromatic, it transmits all the pixel values within this small area directly, without encoding them as an image. The purpose of this design is to reduce the computational resource consumption on both the server and client, thereby lowering the performance load during the operation of remote desktop software.

#### 5.2.3. Expansion of Areas Awaiting Update

Before encoding the partitioned sub-rectangles awaiting update, the DeepTight encoding expands their edges to fit the input size required by deep learning-based image compression encoders. The specific expansion rules are as follows: the width of the sub-rectangle is extended to the right to be a multiple of 16, using the rightmost column of pixels from the unexpanded sub-rectangle as the fill pixels. Then, the length of the sub-rectangle is increased downwards to be a multiple of 16, using the bottom row of pixels from the right-extended sub-rectangle as the fill pixels for expansion. The algorithm for expanding the update areas is as follows (Algorithm 3):
**Algorithm 3** Rectangle Expansion**Require:** The pixels of an unexpanded rectangle ur with its left-top pixel location (left,top) and its right-bottom pixel location (right,bottom);**Ensure:** An expanded rectangle er;  1:W←round((right−left+16−1)/16)×16  2:H←round((bottom−top+16−1)/16)×16  3:**for** j←top to (top+H) **do**  4:   **for** i←left to (left+W) **do**  5:      **if** j<top **then**  6:         **if** i<right **then**  7:            er(i,j)←ur(i,j)  8:         **else**  9:            er(i,j)←ur(right,j)10:         **end if**11:      **else**12:         er(i,j)←ur(i,bottom)13:      **end if**14:   **end for**15:**end for**16:**return** er

#### 5.2.4. Explanation of Encoded Data Format

The Deep encoding uses 8 bytes, or 64 bits, to describe the position information of a specific area awaiting update. This includes 32 bits to describe the position of the top-left pixel of the update area and another 32 bits for the bottom-right pixel position. Additionally, 4 bytes, or 32 bits, are required to describe the encoding method used for the update area. The header format for the update area is as depicted in Table 2, where the encoding format number for Deep encoding is designated as 24.

After detailing the position information and encoding method of the area awaiting update, Deep encoding requires further elaboration on the encoding of the update area based on its own specifics. Therefore, following the header information of the update area, there is an additional header for Deep encoding to facilitate the client’s processing of Deep encoded content. Table 3 lists the header format for Deep encoding.

Following the header information for Deep encoding is the area awaiting update that will be processed with Deep encoding. The client can correctly receive the data for the update area based on the amount of entropy-encoded data specified in the header. The verification of the received Deep encoded update area is performed by using the checksum and the data volume before entropy encoding, as indicated in the header. This allows for the correct decompression of the update area, after which the decompressed data are passed to the image decoder for decoding and filling the sub-rectangles.

As Deep encoding involves the re-partitioning of areas awaiting update, each subdivided sub-rectangle requires its own header information to record relevant details before and after encoding. Table 4 lists the header information for a single sub-rectangle.

## 6. Experiments and Analysis

**Figure 4:**
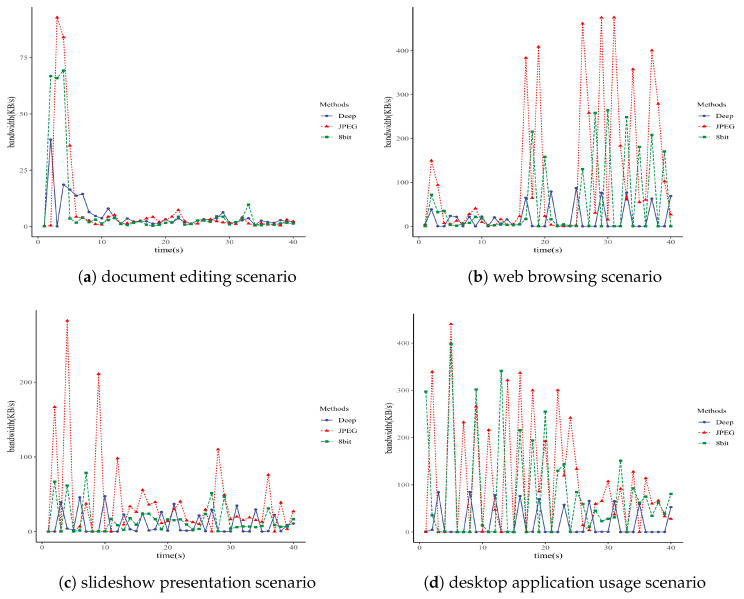
Bandwidth–time curves in different scenarios.

In this paper, the remote desktop clients used in the experiments were deployed on two Windows 10 computers with x86 architecture, equipped with Intel i9-10900 CPUs and 64GB of memory. This experiment analyzed bandwidth-time curves for three RFB protocol encoding methods: DeepTight encoding based on deep image compression, Tight encoding using the JPEG image compression algorithm, and the 8-bit mode provided by VNC. The Figure 4 show that, in the same scenarios, the DeepTight encoding proposed in this paper consumed less network bandwidth than the other two low-bandwidth encoding methods available in VNC for most of the time. Table 5 lists detailed bandwidth data for these three encoding methods in scenarios such as document editing, web browsing, slideshow presentation, and desktop application usage. The data reveals that, across all four scenarios, DeepTight encoding uses less average and peak bandwidth compared to the other two methods. Specifically, compared to Tight encoding with JPEG compression, DeepTight encoding saved 34.08%, 84.60%, 72.96%, and 83.89% of average bandwidth and 58.42%, 81.68%, 83.19%, and 80.78% of peak bandwidth in the four scenarios, respectively. In comparison to the 8-bit color depth method, DeepTight encoding saved 29.80%, 66.67%, 32.18%, and 77.99% of average bandwidth and 44.27%, 67.03%, 39.64%, and 78.73% of peak bandwidth in each respective scenario. Average bandwidth represents the network traffic consumption of a remote desktop protocol. Generally, the lower the average bandwidth required by a remote desktop protocol over a period of time, the less the total network traffic consumption. Peak bandwidth determines the maximum network bandwidth demand of the remote desktop protocol. The lower the peak bandwidth required by the remote desktop protocol, the lower the network bandwidth needed for smooth operation. The data shows that, in terms of both network traffic and bandwidth, DeepTight requires the least, making it more adaptable to low-bandwidth environments compared to the other two methods.

**Figure 5:**
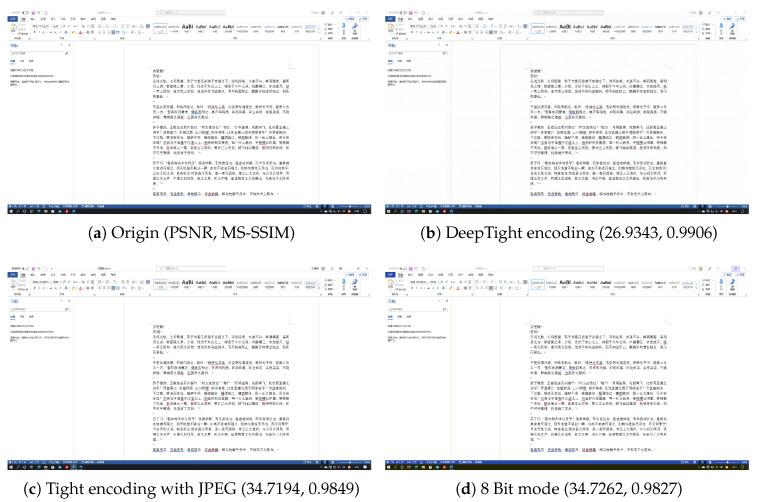
The comparisons of desktop images in a document editing scenario.

**Figure 6:**
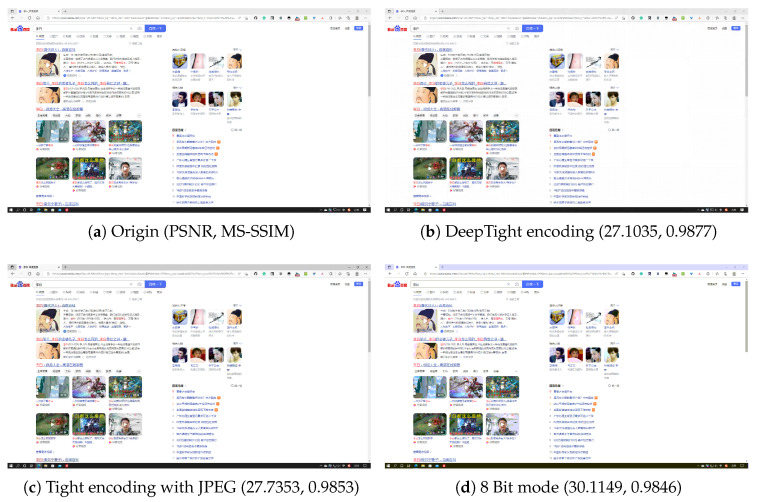
The comparisons of desktop images in a Web surfing scenario.

**Figure 7:**
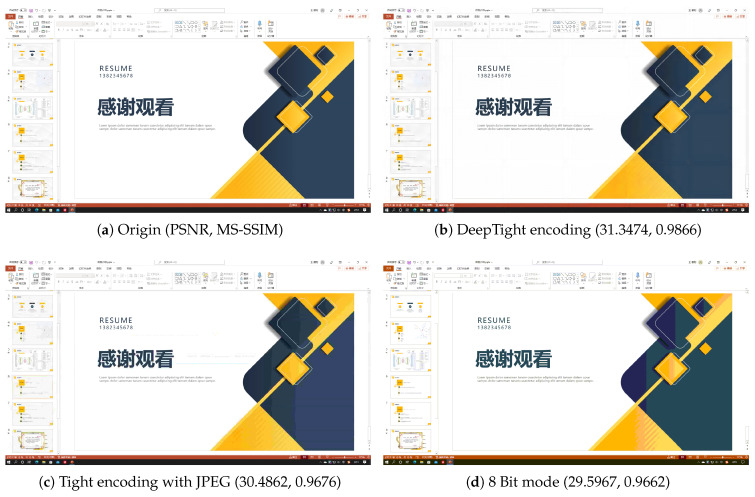
The comparisons of desktop images in a Microsoft PowerPoint scenario.

**Figure 8:**
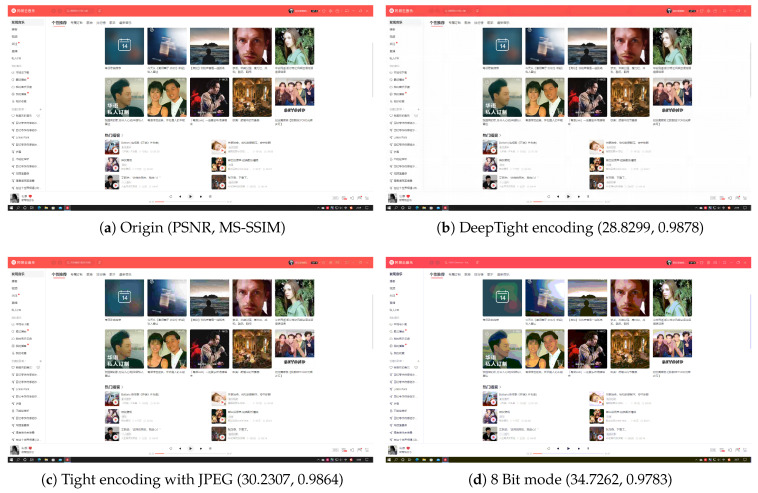
The comparisons of desktop images in using a desktop application scenario.

Figure 5, Figure 6, Figure 7 and Figure 8 each demonstrate the reconstructed desktop images and their level of distortion relative to the original server-side desktop images under different encoding schemes in four scenarios: document editing, web browsing, slideshow presentation, and desktop application usage. Table 6 provides a detailed display of the PSNR and MS-SSIM scores for different encodings in four scenarios. The data reveal that in all these scenarios, DeepTight encoding surpasses the other two encoding methods in terms of MS-SSIM scores for reconstructed desktop images. In PSNR comparison, however, DeepTight encoding outperforms the others only in the slideshow presentation scenario, while in the remaining scenarios, the scores for DeepTight encoding’s reconstructed images are comparable to or slightly lower than those of the other two encoding methods. PSNR represents the average difference between the reconstructed image and the original image. In the other three scenarios, there is a significant amount of whitespace in the desktop images. DeepTight encoding’s restoration of these whitespace areas is not as effective as the other two methods. However, these whitespace areas are non-working zones that users generally do not focus on. Minor distortions in these areas are unlikely to impact the users’ actual visual experience. From the graphical analysis, it can be observed that compared to Tight encoding, DeepTight encoding restores desktop images with greater color continuity and smoothness. In contrast to the 8 Bit mode, DeepTight encoding achieves more accurate color reproduction. Moreover, among all encoding methods, DeepTight encoding consumes the least bandwidth, both in terms of average and peak usage.

Additionally, in all four scenarios, compared to Tight encoding using the JPEG compression algorithm, DeepTight encoding effectively reduces block artifacts and shadow effects in the reconstructed desktop images. In contrast to encoding methods using 8-bit color depth, DeepTight encoding more accurately restores the colors of the original server-side desktop images and also reduces block artifacts resulting from lower pixel color depth.

## 7. Conclusions

This study advances convolutional neural networks, introducing an end-to-end model for image compression and decompression with corresponding computational equations. It also enhances the RFB protocol, a key component in VNC’s remote transmission, by integrating a novel scheme, Deep encoding, which is tailored for deep image compression and decompression codecs. This innovation led to the development of a remote desktop prototype using open-source VNC code based on the Deep encoding approach.

This paper details an image compression and decompression model fine-tuned for optimal human perception of reconstructed image quality. It seamlessly integrates adaptive spatial and channel attention mechanisms, comprising an encoder, a decoder, and a hyperprior codec. The encoder extracts latent image features, the decoder reconstructs images from these features, and the hyperprior codec converts these features into Gaussian form, aiding in achieving an improved rate–distortion equilibrium.

For optimization, the study employs Mean Squared Error (MSE) and Multi-Scale Structural Similarity (MS-SSIM) as combined metrics to assess image reconstruction distortion. The findings reveal that this dual-metric approach not only boosts MS-SSIM performance in desktop image reconstruction but also retains greater structural and textural fidelity compared to MSE-only optimizations, which can smooth out important textural nuances. This paper further introduces an attention mechanism within the model, targeting latent feature representations across both channel and spatial dimensions, thereby enhancing rate–distortion effectiveness. Testing shows that this addition elevates Peak Signal-to-Noise Ratio (PSNR) performance while preserving MS-SSIM quality.

To adapt this model for the open-source VNC remote desktop, this paper introduces Deep encoding—a fresh approach to remote desktop transmission encoding. This method, focused on segmenting, identifying, and encoding diverse image feature-based update areas, marks a significant refinement of the RFB protocol. The prototype, built on Deep encoding and VNC code, was evaluated against two low-bandwidth VNC encoding schemes, demonstrating up to 84.60% average and 83.19% peak bandwidth savings. Its MS-SSIM performance surpasses all existing low-bandwidth image encoding methods in VNC. Moreover, images reconstructed with Deep encoding show markedly fewer block effects and artifacts in low-bandwidth scenarios, substantially improving the user experience.

## Figures and Tables

**Table 1:** Desktop image encoding methods in the RFB protocol.

Code	Name
0	Raw
1	CopyRect
2	RRE
4	CoRRE
5	Hextile
16	ZRLE
6	zlib
7	Tight
8	zlibhex

**Table 2:** Header of an update-rectangle.

Length (Bytes)	Type	Variable Name	Description
2	Unsigned Short	x	X-coordinate of the update area
2	Unsigned Short	y	Y-coordinate of the update area
2	Unsigned Short	w	Width of the update area
2	Unsigned Short	h	Height of the update area
4	Unsigned Int	encoding	Encoding format description

**Table 3:** Header of Deep encoding.

Length (Bytes)	Type	Variable Name	Description
4	Unsigned Int	block_num	Number of sub-rectangles in the area
4	Unsigned Int	data_size	Compressed data size (in bytes)
4	Unsigned Int	origin_size	Original data size (in bytes) before compression
4	Unsigned Int	checksum	Checksum

**Table 4:** Header of a sub-rectangle.

Length (Bytes)	Type	Variable Name	Description
2	Unsigned Short	x	Sub-rectangle position
2	Unsigned Short	y	Sub-rectangle position
2	Unsigned Short	height	Sub-rectangle height
2	Unsigned Short	width	Sub-rectangle width
2	Unsigned Short	padded_height	Height of the sub-rectangle after padding
2	Unsigned Short	padded_width	Width of the sub-rectangle after padding
4	Unsigned Int	data_size	Size of the encoded data (in bytes)

**Table 5:** The performance data of different encodings in different scenarios.

Encoding Method	Average Bandwidth (KB/s)	Peak Bandwidth (KB/s)	Usage Scenario
DeepTight encoding	4.97	38.61	Document editing
Tight encoding	7.54	92.85	Document editing
8 bit color mode	7.08	69.28	Document editing
DeepTight encoding	17.48	87.18	Web browsing
Tight encoding	113.50	475.80	Web browsing
8 bit color mode	52.47	264.43	Web browsing
DeepTight encoding	10.62	47.45	Slideshow presentation
Tight encoding	39.27	282.27	Slideshow presentation
8 bit color mode	15.66	78.61	Slideshow presentation
DeepTight encoding	17.65	84.61	Using desktop applications
Tight encoding	109.53	440.18	Using desktop applications
8 bit color mode	80.19	397.81	Using desktop applications

**Table 6:** The PSNR and MS-SSIM of different encodings in different scenarios.

Scenario	Encoding	PSNR	MS-SSIM
Document Editing	DeepTight	26.9343	0.9906
Document Editing	Tight	34.7194	0.9849
Document Editing	8 Bit mode	34.7262	0.9827
Web Surfing	DeepTight	27.1035	0.9877
Web Surfing	Tight	27.7353	0.9853
Web Surfing	8 Bit mode	30.1149	0.9846
Power Point	DeepTight	31.3474	0.9866
Power Point	Tight	30.4862	0.9676
Power Point	8 Bit mode	29.5967	0.9662
Desktop Application	DeepTight	28.8299	0.9878
Desktop Application	Tight	30.2307	0.9864
Desktop Application	8 Bit mode	34.7262	0.9783

## Data Availability

The data presented in this study are available on request from the corresponding author on reasonable request.
